# National Trends in Colorectal Cancer Incidence Among Older and Younger Adults in Canada

**DOI:** 10.1001/jamanetworkopen.2019.8090

**Published:** 2019-07-31

**Authors:** Darren R. Brenner, Emily Heer, R. Liam Sutherland, Yibing Ruan, Jill Tinmouth, Steven J. Heitman, Robert J. Hilsden

**Affiliations:** 1 Department of Oncology, Cumming School of Medicine, University of Calgary, Calgary, Alberta, Canada; 2 Department of Cancer Epidemiology and Prevention Research, CancerControl Alberta, Alberta Health Services, Calgary, Alberta, Canada; 3 Department of Community Health Sciences, Cumming School of Medicine, University of Calgary, Calgary, Alberta, Canada; 4 Institute of Health Policy, Management and Evaluation, Department of Medicine, University of Toronto, Toronto, Ontario, Canada; 5 Department of Medicine, Sunnybrook Health Sciences Centre, Toronto, Ontario, Canada; 6 Department of Medicine, Cumming School of Medicine, University of Calgary, Calgary, Alberta, Canada

## Abstract

**Importance:**

Recent evidence has shown that although the incidence of colorectal cancer (CRC) is decreasing among older adults, rates have increased in adults younger than 50 years. Given that younger adults are typically classified as at low risk for the disease, this epidemiologic shift is cause for concern.

**Objective:**

To analyze Canadian national cancer incidence registries to determine incidence trends for CRC among older and younger adults, updated to 2015.

**Design, Setting, and Participants:**

This cohort study determined the incidence of CRC using data from the National Cancer Incidence Reporting System (1969-1992) and the Canadian Cancer Registry (1992-2015). All Canadians diagnosed with CRC from January 1, 1969, through December 31, 2015, were included in this study. Trends among men and women were examined separately and by age category (>50 vs <50 years). Birth cohort models were fit using 5-year groups starting in 1886, with 1936 as the reference cohort. Data were analyzed from May 13, 2018 to May 16, 2018.

**Main Outcomes and Measures:**

Annualized percentage changes and incidence rate ratios of CRC.

**Results:**

From 1971 to 2015, 688 515 incident cases (52.9% women) of CRC were identified. Although the incidence of CRC has decreased in older men and women, rates among younger men and women have increased since 2006 and 2010, respectively. For women younger than 50 years, incidence has increased with a mean annual percentage change of 4.45% since 2010; for men younger than 50 years, a mean annual percentage change of 3.47% from 2006 through 2015. There was an association between CRC incidence and birth cohort, with more recent cohorts being at greater risk than those born earlier. For men, the risk of colorectal cancer in the youngest cohort is more than double that of the reference (incidence rate ratio, 2.57; 95% CI, 1.32-5.02). Incidence rate ratios were not significant for women (IRR, 2.12; 95% CI, 0.95-4.70) but increased in successively younger cohorts.

**Conclusions and Relevance:**

This study found increasing incidence of colorectal cancer diagnoses among Canadian men and women younger than 50 years of age. This increase in incidence among a low-risk population calls for additional research on possible risk factors that may be affecting these younger cohorts. It appears that primary prevention should be the highest priority to reduce the number of younger adults developing CRC in the future.

## Key Points

**Question:**

Is the incidence of colorectal cancer among younger adults still increasing in Canada?

**Findings:**

This cohort study used data from comprehensive Canadian national cancer registries and included all 688 515 incident colorectal cancers diagnosed from 1969 to 2015. The incidence of colorectal cancer among younger adults increased from 2006 to 2015 among men with an annual percentage change of 3.47% and from 2010 to 2015 among women with an annual percentage change of 4.45%.

**Meaning:**

These results provide evidence that the increased incidence of colorectal cancer among younger adults in Canada is continuing and possibly accelerating.

## Teaser

This cohort study analyzes Canadian cancer incidence registries to determine incidence trends for colorectal cancer among older and younger adults.

## Introduction

Colorectal cancer (CRC) is the second most common cancer in Canada, with approximately 26 800 cases diagnosed in 2017.^1^ Incidence of CRC has been declining among adults older than 50 years for several decades. However, a concurrent trend of increasing incidence among adults younger than 50 years has been reported in the United States^2^ and Canada^3^ to 2012. Individuals younger than 50 years are classified as at low risk for CRC and are younger than the recommended screening age, so this increasing incidence is cause for concern. Increases in cancer incidence can sometimes be an artifact of changes in diagnostic or screening practices and can result in detection bias. Although diagnostic practices have undergone some changes, including an increase in colonoscopy use for nonscreening purposes,^4^ these changes cannot explain most of the new cases. More likely, lifestyle factors associated with increased weight gain are fueling the rise in CRC incidence in this age group.^5^

Brenner et al^3^ previously published an analysis demonstrating increased incidence rates in younger adults for colon and rectal cancers with data to 2012. The objective of the present study is to investigate whether the trends observed previously have continued past 2012. Herein we present an update to the previous analyses using comprehensive national incidence data in Canada from January 1, 1969, through December 31, 2015, with an additional analysis by birth cohort.

## Methods

### Registries and Coding Classifications

Complete historical incidence data for colon cancer (codes C18.0, C18.2-18.9, and C26.0 from *International Statistical Classification of Diseases and Related Health Problems, Tenth Revision* [*ICD-10*]) and rectal cancer (codes C19 and C20 from *ICD-10*) were collected from the National Cancer Incidence Reporting System (1969-1992) and the Canadian Cancer Registry (1992-2015) in May 2018. The Canadian Cancer Registry maintains and collects data on cancer cases that are submitted by individual registries in Canadian provinces and territories. Reporting cancer cases is a legislated responsibility of the provinces and territories, which ensures high-quality reporting. This analysis was approved by the Health Research Ethics Board of Alberta, which did not require informed consent for the use of publicly available data. This study adheres to the reporting guidelines outlined in the Strengthening the Reporting of Observational Studies in Epidemiology (STROBE) reporting guideline.^6^

### Statistical Analysis

Data were analyzed from May 13, 2018 to May 16, 2018. Trends were examined among men and women and by age group (<50 vs ≥50 years) for combined colon and rectal cancer incidence. Annualized percentage changes (APCs) in incidence rate were estimated using the Joinpoint Regression Program, version 4.5.0.1 (National Cancer Institute), as described in a previous publication.^3^ Briefly, Joinpoint regression models were fit to log-transformed incidence rates, and permutation analysis was used to select the best-fit model ranging from 0 to 4 Joinpoints. Birth cohort models were fit using the National Cancer Institute’s web tool,^7^ which quantifies the association of age, period, and cohort using age-drifted–period–cohort models. Input data were cases and population for 14 five-year age groups and 8 five-year periods. Cohort effects are presented as incidence rate ratios (IRRs) with 1936 as the reference birth cohort, with adjustments for age. The first cohort included in this analysis was born in 1886, and the most recent cohort was born in 1986. All statistical tests used a level of significance at 2-sided α = .05.

## Results

### Incidence Trends of CRC

**Figure 1.**
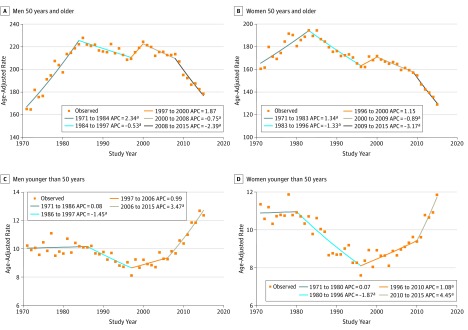
Observed Incidence Trends of Colorectal Cancer by Sex and Age in Canada Annual percentage changes (APCs) are given for each birth year range. ^a^Indicates that the APC is significantly different from zero at α = .05 level.

From 1971 to 2015, a total of 688 515 incident cases of CRC were identified, including 363 895 men (47.1%) and 324 620 women (52.9%). The individual cohort data can be found in the eTable in the Supplement. The incidence of CRC has distinct and opposite trends for older vs younger adults. For older men, the rate of CRC increased from 1971 to 1984 by an APC of 2.34%. From 1984 to 1997, incidence decreased by an APC of −0.53% (Figure 1A). Women older than 50 years had a similar trend, with an APC of 1.34% from 1971 to 1983, and decreasing rates by a mean APC of −1.33% from 1983 to 1996 (Figure 1B). Rates then increased for men and women until 2000 with APCs of 1.87% and 1.15%, respectively. Incidence of CRC then had an uninterrupted decrease until 2015. Rates were consistently lower among women, with the age-adjusted rate peaking at approximately 192 per 100 000 compared with the peak for men at 225 per 100 000.

Incidence rates of CRC incidence for younger individuals were largely unchanged from 1970 to 1986 for men (Figure 1C) and from 1971 to 1980 for women (Figure 1D). After 1986, incidence among younger men decreased by an APC of −1.45% to 1997. Among women, rates decreased until 1996 by an APC of −1.87%. Rates then increased among men and women to 2015. Among younger men, incidence increased by a mean APC of 3.47% from 2006 to 2015; for younger women, the mean APC was 4.45% from 2010 to 2015. In 1971, incidence of CRC among men younger than 50 years was approximately 10 per 100 000 compared with an incidence of approximately 12.5 per 100 000 in 2015. Similarly, the incidence among younger women in 1971 was just less than 11 per 100 000, whereas it rose to approximately 12 per 100 000 in 2015.

Incidence rates were also examined by site and age group. Rates of colon cancer among women (eFigure 1 in the Supplement) and men (eFigure 2 in the Supplement) aged 20 to 39 years increased dramatically to 2015. For women, rates increased from 2001 to 2015 in the group aged 20 to 29 years and from 1998 to 2015 in the group aged 30 to 39 years. Among men, the significant increases occurred from 1995 to 2015 in the group aged 20 to 29 years and from 2000 to 2015 in the group aged 30 to 39 years. However, significant increases were also seen in the groups aged 40 to 49 years for both sexes. Significant recent decreases were also seen in every age group older than 50 years for men and women. The most substantial decrease occurred in the oldest group (80-89 years), with rates dropping to the lowest observed for men and women.

The incidence of rectal cancer is much lower than that of colon cancer, but significant changes were still observed in the 10-year age groups. Rates have been rising steadily in the youngest group for women (eFigure 3 in the Supplement), whereas no significant change was observed in this group for men (eFigure 4 in the Supplement). Women younger than 50 years had significant increases in incidence in every age group. A sharp and significant increase in incidence has been seen among men aged 30 to 39 years since 2003 and in the group aged 40 to 49 years since 1999. Significant decreases in incidence were observed for women aged 70 to 89 years and for men aged 60 to 89 years.

### Birth Cohort Analysis

**Figure 2.**
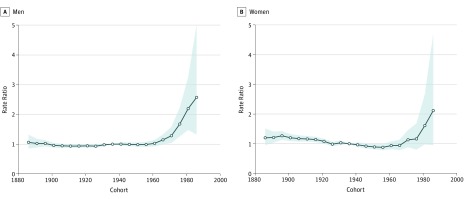
Incidence Rate Ratios for Colorectal Cancer in Men and Women by Birth Cohort Every 5-year interval indicates 1 cohort from 1886 to 1986, with the 1936 cohort as the reference group. Incidence data were collected from the National Cancer Incidence Reporting System (1969-1992) and the Canadian Cancer Registry (1992-2012). Shaded areas indicate 95% CIs of the estimated incidence rate ratios.

Analysis by birth cohort also revealed a strong effect, with more recent cohorts having higher rates of CRC than those born earlier. For men, an increase in the incidence rate was noted from the 1966 birth cohort (IRR, 1.14; 95% CI, 0.99-1.33), and the rates rose continuously in the younger birth cohorts up to 1986 (IRR, 2.57; 95% CI, 1.32-5.02) (Figure 2A). For women, the increase in incidence rates was not statistically significant. Nevertheless, the IRR increased from 1.16 (95% CI, 0.80-1.69) in the 1976 birth cohort to 1.61 (95% CI, 0.98-2.66) in the 1981 birth cohort and 2.12 (95% CI 0.95-4.70) in the 1986 birth cohort (Figure 2B). The most recent cohort for men and women had more than a 2 times higher risk of developing CRC than the reference cohort of 1936. In men and women, those born in the most recent cohorts (post-1980s) have the highest rates on record. Before these dates, rates of CRC were similar among birth cohorts.

## Discussion

The results of these analyses confirm the presence of an association between birth cohort and increasing incidence of CRC among individuals younger than 50 years. The addition of 2015 data provides evidence of the continuance and potential acceleration of the trend reported previously.^3,4^ Together these data suggest a common exposure in North America that may be placing younger adults at higher risk of CRC compared with those born earlier. An association with birth cohort also suggests early life exposure or an exposure that accumulates during the life course.^8^

A previous report^3^ described the association of screening and obesity with these observed trends. After the implementation of Canadian guidelines recommending colonoscopy screening for adults older than 50 years, incidence of colon and rectal cancer among adults in this age group declined significantly. Slope changes after 2004 were −1.85 per 100 000 for colon cancer and −0.66 per 100 000 for rectal cancer. No significant changes occurred among adults younger than 50 years after colonoscopy guidelines were implemented in Canada. Colorectal cancer is typically diagnosed in older adults in high-income countries, and young adult–onset CRC is usually connected to a family history of the cancer or Lynch syndrome.^9^ However, given the recent and dramatic changes in incidence, the rising prevalence of obesity in high-income countries is a possible driver for the increasing CRC trends among younger adults.

After the reports of increased CRC incidence among younger adults in the United States, the American Cancer Society updated their screening eligibility to include average-risk individuals starting at 45 years of age.^10^ The rationale for reducing the age of screening eligibility was that the increased incidence in the younger population was unlikely to be the result of factors that may induce bias.^10^ Although use of colonoscopies for procedures other than CRC screening has grown, authors of the recommendations state that the practice is not widespread enough in the younger cohort to cause detection bias. In addition, mortality rates have increased in some demographic groups, which suggests that the rise in incidence has not been among those who are diagnosed at an earlier stage of disease.^11^ Finally, the presence of a birth cohort effect in the US data does not support the detection bias hypothesis. The successive increase in risk with each new birth cohort suggests a continuing trend and not the effect of a singular change in medical or diagnostic practices.

Canadian guidelines have remained unchanged owing to a lack of evidence to substantiate any recommendations and the relatively low number of cases in this younger group. It appears that analyses should be conducted on trends in CRC mortality by age and on colonoscopy practices in Canada for procedures other than cancer screening. However, the heightened risk in the youngest birth cohorts demonstrated by our models suggest there is evidence of a true increase in incidence. We believe that the data presented herein, which to our knowledge are the most up-to-date in North America, provide a strong rationale for additional research in 3 key areas to guide any decisions to alter screening recommendations in single-payer systems such as Canada.

First, we believe that more research is needed into the risk factors associated with the epidemiologic placing of younger individuals at greater risk. Second, further research should be performed among these younger groups to potentially guide targeted screening programs. The current analysis shows that individuals in the group aged 40 to 49 years are driving the increased risk in the younger population, with most of the cases younger than 50 years occurring in this group. This indicates that the revised screening guidelines starting at 45 years of age, similar to those from the United States, might target the most relevant age group in terms of increasing incidence. Third, we believe that analyses on the effects of changing recommendations are needed, particularly on how changing screening guidelines would affect incidence and mortality. Based on these figures, it appears we should determine whether screening younger individuals would be cost-effective, as was recently investigated by a group in Australia.^12^ The implications of changing screening guidelines also need to be better understood. Although reducing the age of screening onset may result in decreased mortality of younger individuals, several possible negative consequences need to be explored in more detail.^13^ Colonoscopy screening for younger populations may be needlessly invasive, may divert resources from individuals at higher risk, and could increase colonoscopy wait times, particularly if messaging is unclear and younger patients are erroneously referred for colonoscopies.

To aid in the decision-making process of whether to change screening eligibility criteria in Canada, we recommend that further research be conducted in the following areas. First, our findings suggest that the performance characteristics of screening tools within the younger screening population should be studied. To date, the sensitivity and specificity of fecal-based tests have been largely limited to older adults,^14^ which may not be generalizable. These findings would help to justify the use of additional resources for adults younger than 50 years. Second, the risk factors for CRC in this younger cohort should be further studied to determine appropriate strategies for primary prevention. As described previously, obesity levels are likely driving this increased risk among younger adults.^3^ We believe prevention of this risk factor should be a priority of public health efforts, while other possible etiologic factors that may be driving the increase in incidence should also be studied.

### Limitations

Although this study addresses an issue of public health concern, the limitations should be addressed. Owing to the nature of the registry data, we did not have access to any demographic information except age and thus could not identify whether any other factors could be associated with these incidence trends. We also did not have access to histologic information to address whether trends were limited to specific histology groups.

## Conclusions

Given this evidence of rising incidence in a group once considered to be at low risk, we believe that steps need to be taken in Canada to reduce the number of young people with CRC in the future and that primary prevention should remain the highest priority for future research. The reasons for this increase are not well understood, but we believe they should be investigated before a change in current screening guidelines is pursued. The observed association between CRC incidence and birth cohort in this analysis adds salience to this public health problem because it suggests that increasing numbers of younger adults will develop CRC in the coming decades.

## Notes

Supplement. **eTable.** Number of Incident Colorectal Cancer Cases From 1971 to 2015 **eFigure 1.** Age-Specific Incidence Rates of Colon Cancer Among Women in Canada (1971-2015) by 10-Year Age Groups **eFigure 2.** Age-Specific Incidence Rates of Colon Cancer Among Men in Canada (1971-2015) by 10-Year Age Groups **eFigure 3.** Age-Specific Incidence Rates of Rectal Cancer Among Women in Canada (1971-2015) by 10-Year Age Groups **eFigure 4.** Age-Specific Incidence Rates of Rectal Cancer Among Men in Canada (1971-2015) by 10-Year Age Groups
