# Supplementary material for: National Trends in Colorectal Cancer Incidence Among Older and Younger Adults in Canada
Source: JAMA Netw Open. 2019 Jul 31;2(7):e198090. doi: 10.1001/jamanetworkopen.2019.8090 (PMC6669779; doi:10.1001/jamanetworkopen.2019.8090)
Supplement: Supplement. — eTable. Number of Incident Colorectal Cancer Cases From 1971 to 2015 eFigure 1. Age-Specific Incidence Rates of Colon Cancer Among Women in Canada (1971-2015) by 10-Year Age Groups eFigure 2. Age-Specific Incidence Rates of Colon Cancer Among Men in Canada (1971-2015) by 10-Year Age Groups eFigure 3. Age-Specific Incidence Rates of Rectal Cancer Among Women in Canada (1971-2015) by 10-Year Age Groups eFigure 4. Age-Specific Incidence Rates of Rectal Cancer Among Men in Canada (1971-2015) by 10-Year Age Groups [file jamanetwopen-2-e198090-s001.pdf]

Supplementary Online Content

Brenner DR, Heer E, Sutherland RL, et al. National trends in colorectal cancer incidence among older and younger adults in Canada. *JAMA Netw Open.* 2019;2(7):e198090. doi:10.1001/jamanetworkopen.2019.8090

eTable. Number of Incident Colorectal Cancer Cases From 1971 to 2015

eFigure 1. Age-Specific Incidence Rates of Colon Cancer Among Women in Canada (1971-2015) by 10-Year Age Groups

eFigure 2. Age-Specific Incidence Rates of Colon Cancer Among Men in Canada (1971-2015) by 10-Year Age Groups

eFigure 3. Age-Specific Incidence Rates of Rectal Cancer Among Women in Canada (1971-2015) by 10-Year Age Groups

eFigure 4. Age-Specific Incidence Rates of Rectal Cancer Among Men in Canada (1971-2015) by 10-Year Age Groups

This supplementary material has been provided by the authors to give readers additional information about their work.

© 2019 Brenner DR et al. *JAMA Network Open.*

eTable. Number of Incident Colorectal Cancer Cases From 1971 to 2015

		Colon			Rectum			Colorectal	
**Year**	**Male**	**Female**	**Both**	**Male**	**Female**	**Both**	**Male**	**Female**	**Both**
1971	2240	2760	5000	1550	1055	2605	3760	3830	7590
1972	2295	2870	5165	1515	1085	2600	3805	3955	7760
1973	2605	3245	5850	1615	1210	2825	4235	4445	8680
1974	2585	3195	5780	1545	1200	2745	4145	4355	8500
1975	2650	3195	5845	1630	1235	2865	4295	4420	8715
1976	2965	3415	6380	1745	1245	2990	4690	4660	9350
1977	3100	3675	6775	1835	1415	3250	4935	5080	10015
1978	3320	3965	7285	1945	1480	3425	5260	5440	10700
1979	3325	3990	7315	1965	1560	3525	5305	5535	10840
1980	3325	4005	7330	1910	1450	3360	5245	5430	10675
1981	3690	4165	7855	2100	1555	3655	5825	5730	11555
1982	3745	4365	8110	2165	1605	3770	5940	5960	11900
1983	4000	4530	8530	2220	1730	3950	6220	6265	12485
1984	4160	4600	8760	2295	1675	3970	6450	6260	12710
1985	4345	4830	9175	2410	1825	4235	6780	6660	13440
1986	4365	4765	9130	2390	1765	4155	6775	6525	13300
1987	4450	4915	9365	2480	1740	4220	6945	6665	13610
1988	4675	4815	9490	2465	1795	4260	7145	6590	13735
1989	4625	4895	9520	2555	1795	4350	7185	6670	13855
1990	4795	4995	9790	2595	1875	4470	7375	6875	14250
1991	4855	5085	9940	2685	1790	4475	7530	6875	14405
1992	5125	5240	10365	2850	1860	4710	7950	7100	15050
1993	4945	5340	10285	2875	1940	4815	7815	7285	15100
1994	5325	5345	10670	2820	1955	4775	8145	7295	15440
1995	5210	5355	10565	2910	1910	4820	8115	7255	15370
1996	5260	5325	10585	2900	1905	4805	8165	7240	15405
1997	5370	5450	10820	2975	1995	4970	8345	7455	15800
1998	5715	5920	11635	3135	2150	5285	8850	8060	16910
1999	5890	5920	11810	3280	2115	5395	9180	8035	17215
2000	6185	6180	12365	3475	2235	5710	9675	8440	18115
2001	6110	6165	12275	3680	2300	5980	9805	8470	18275
2002	6300	6205	12505	3705	2390	6095	10005	8580	18585
2003	6280	6210	12490	3640	2440	6080	9930	8655	18585
2004	6490	6560	13050	3930	2405	6335	10440	8970	19410
2005	6765	6615	13380	3935	2485	6420	10710	9095	19805
2006	6870	6595	13465	4020	2480	6500	10905	9075	19980

© 2019 Brenner DR et al. *JAMA Network Open.*

2007	7135	6780	13915	4145	2600	6745	11290	9400	20690
2008	7255	6905	14160	4410	2630	7040	11650	9550	21200
2009	7345	6885	14230	4410	2770	7180	11750	9660	21410
2010	7115	7040	14155	4310	2700	7010	11425	9725	21150
2011	7225	6765	13990	4415	2650	7065	11630	9425	21055
2012	7205	6800	14005	4465	2675	7140	11715	9475	21190
2013	7460	6750	14210	4630	2690	7320	12105	9450	21555
2014	7570	6710	14280	4590	2745	7335	12205	9435	21640
2015	7640	6475	14115	4580	2780	7360	12245	9265	21510
1971-2015	229905	235810	465715	133700	88890	222590	363895	324620	688515

© 2019 Brenner DR et al. *JAMA Network Open.*

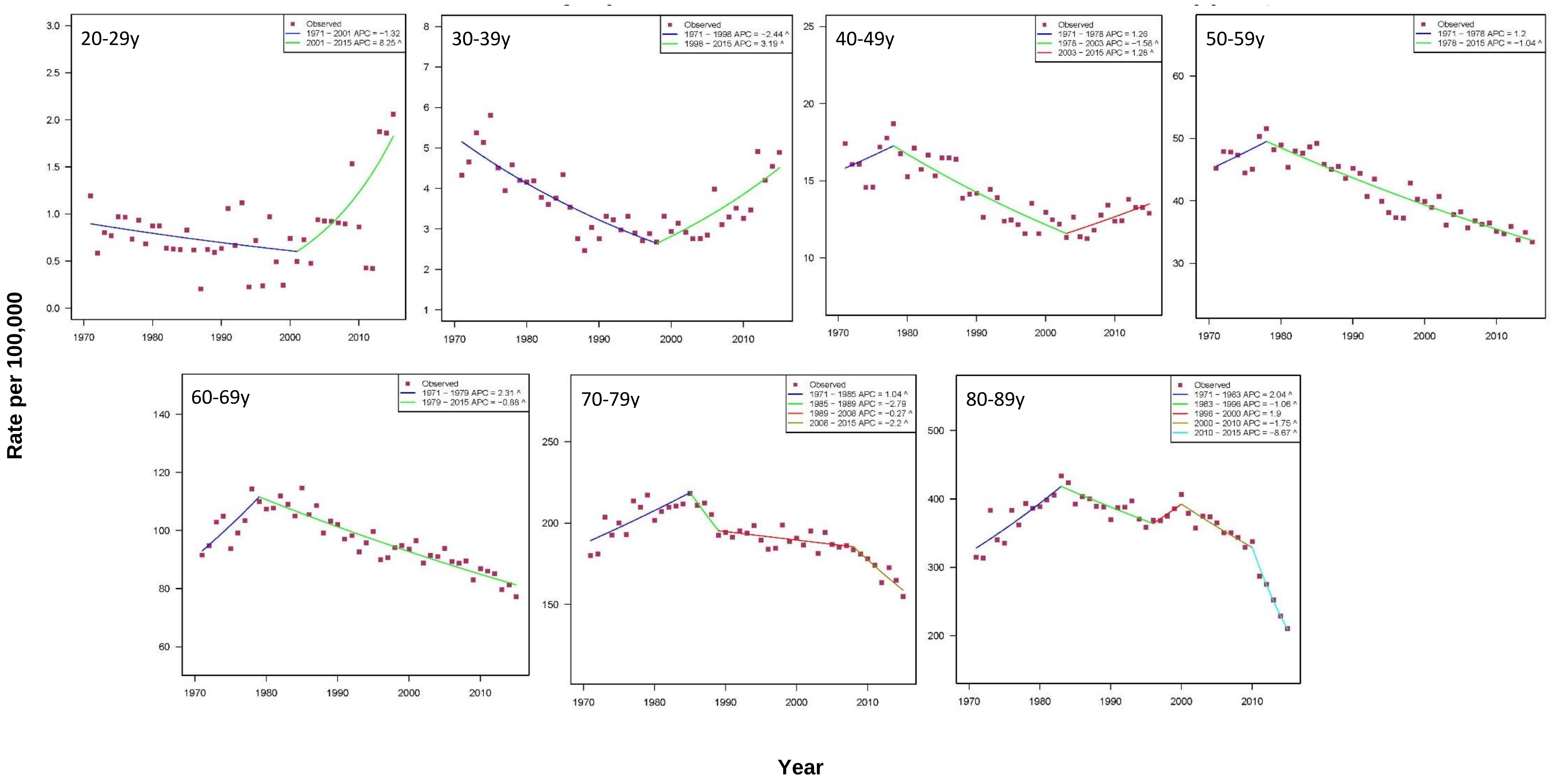

eFigure 1. Age-Specific Incidence Rates of Colon Cancer Among Women in Canada (1971-2015) by 10-Year Age Groups
Annual percent changes given in legend.
^ Indicates that the annual percent change is significantly different from zero at the alpha=0.05 level.

© 2019 Brenner DR et al. *JAMA Network Open.*

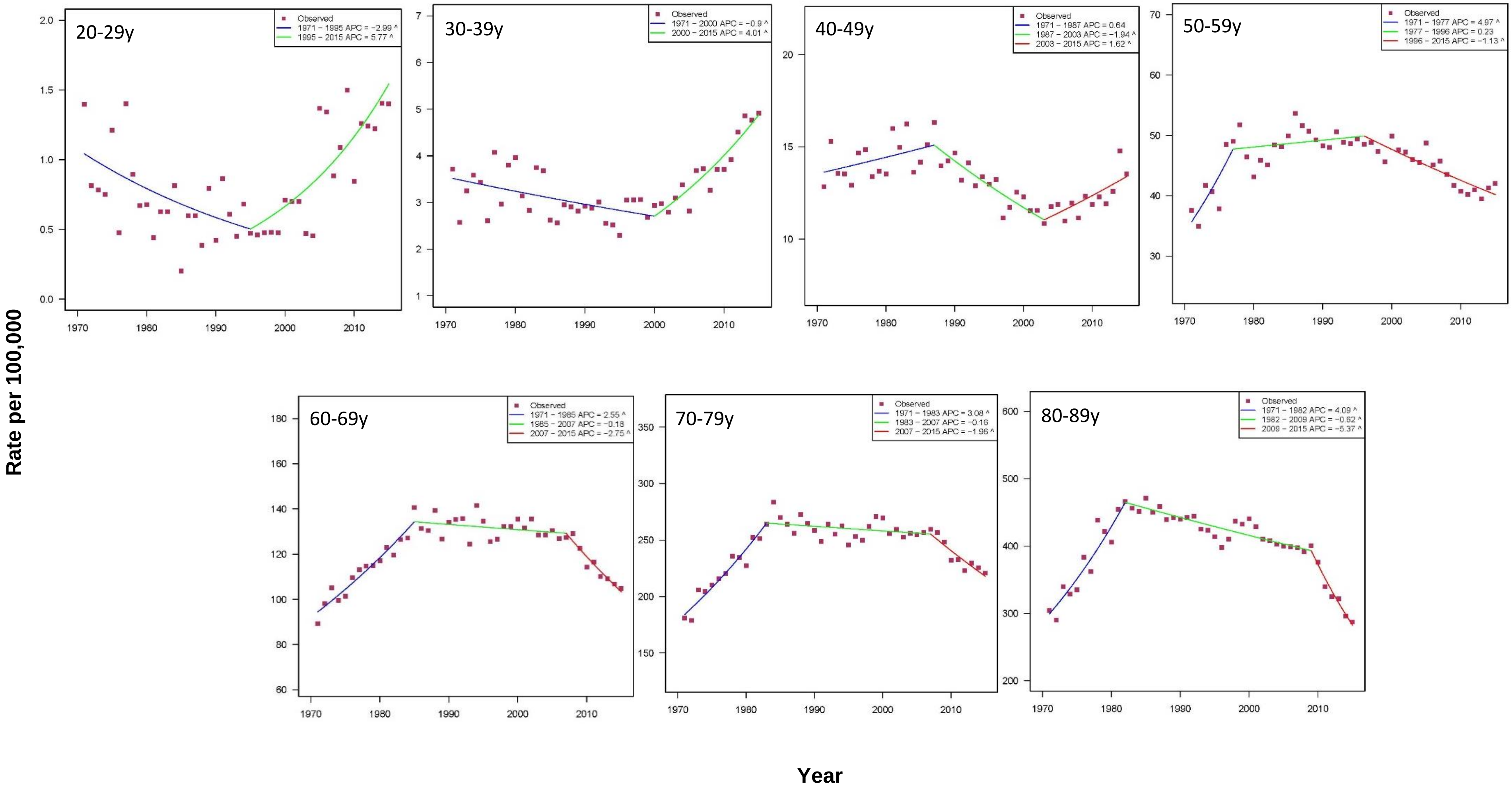

eFigure 2. Age-Specific Incidence Rates of Colon Cancer Among Men in Canada (1971-2015) by 10-Year Age Groups

Annual percent changes given in legend.

^ Indicates that the annual percent change is significantly different from zero at the alpha=0.05

© 2019 Brenner DR et al. *JAMA Network Open.*

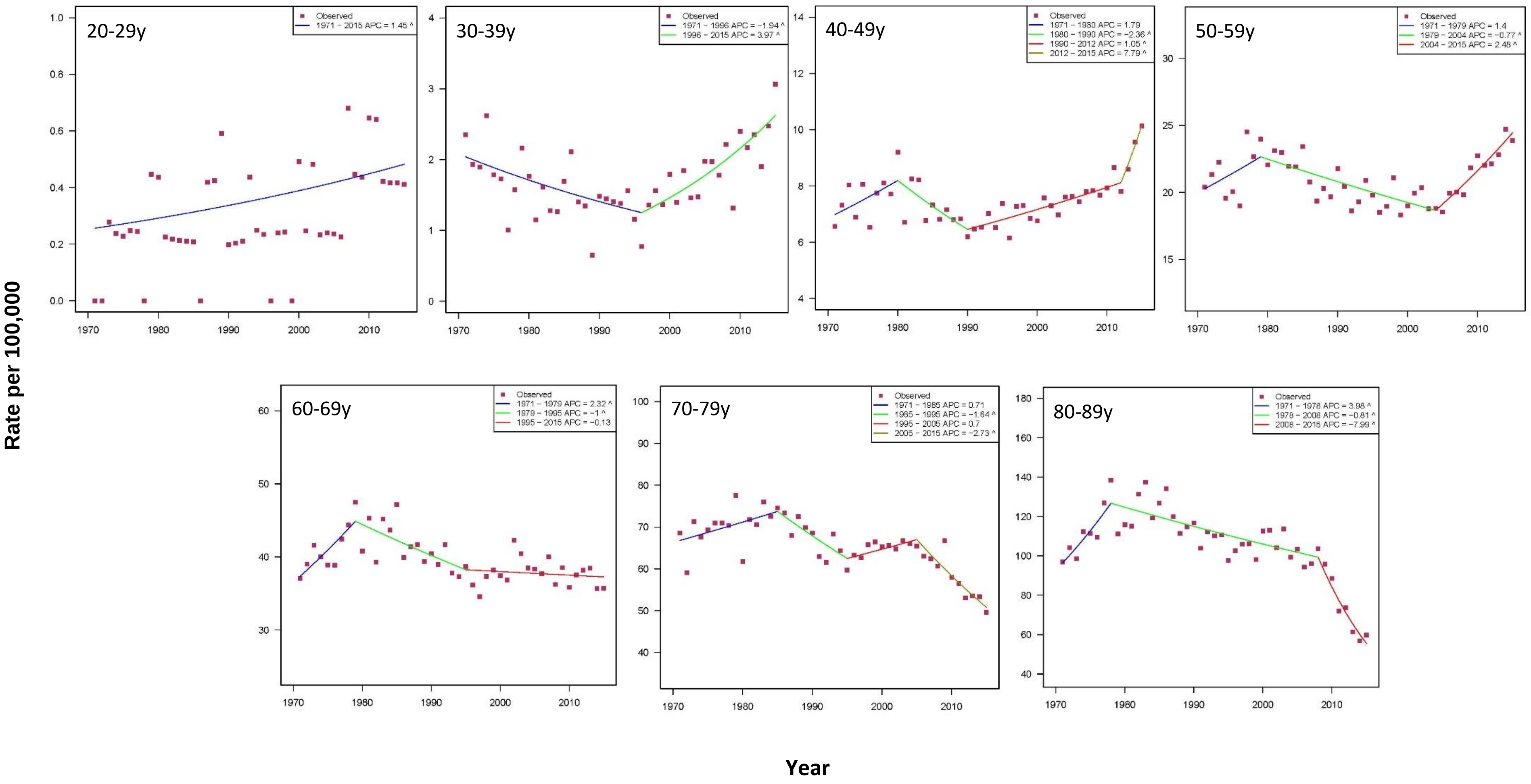

eFigure 3. Age-Specific Incidence Rates of Rectal Cancer Among Women in Canada (1971-2015) by 10-Year Age Groups

Annual percent changes given in legend.

^ Indicates that the annual percent change is significantly different from zero at the alpha=0.05

© 2019 Brenner DR et al. *JAMA Network Open.*

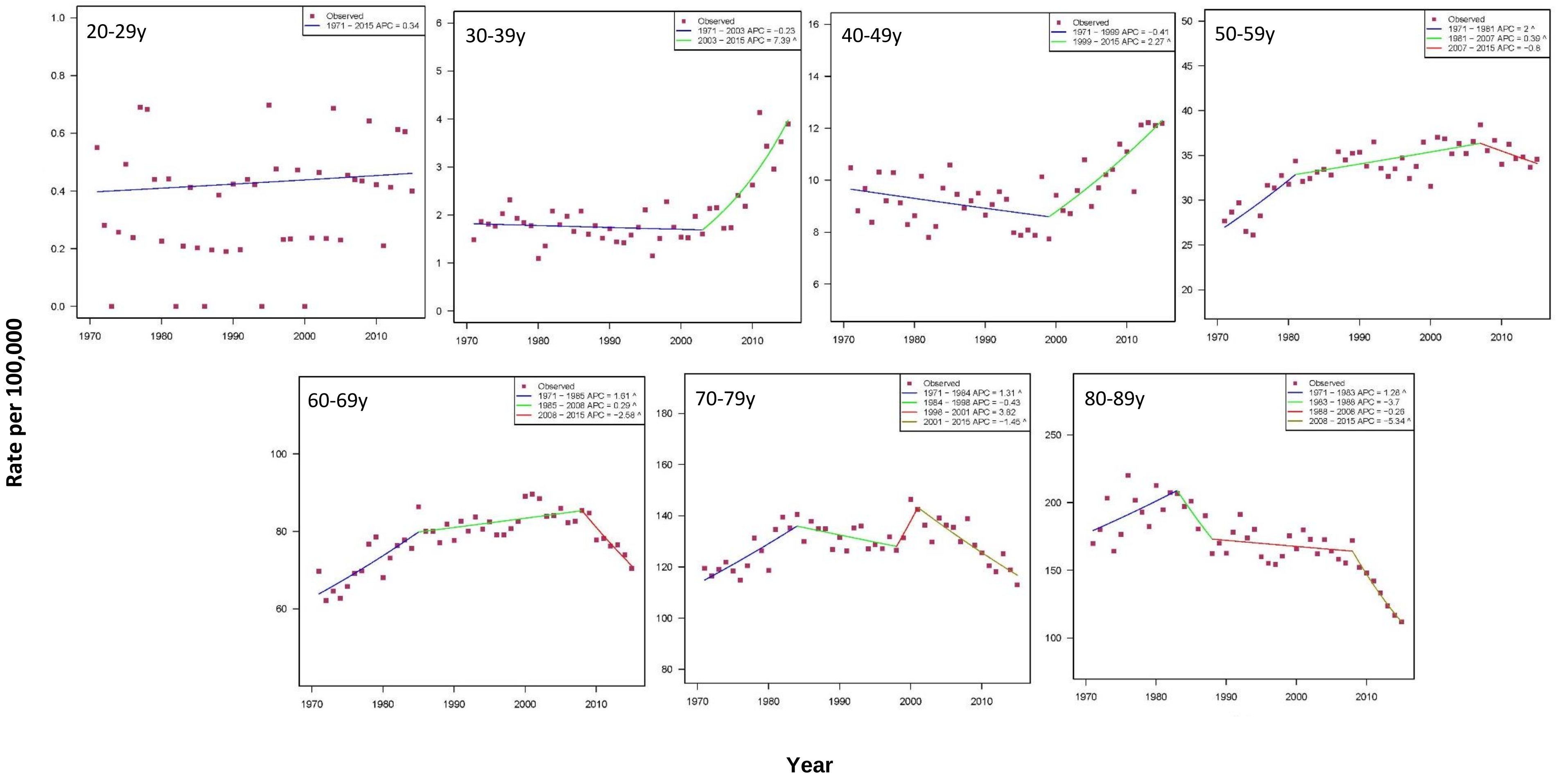

eFigure 4. Age-Specific Incidence Rates of Rectal Cancer Among Men in Canada (1971-2015) by 10-Year Age Groups

Annual percent changes given in legend.

^ Indicates that the annual percent change is significantly different from zero at the alpha=0.05 level

© 2019 Brenner DR et al. *JAMA Network Open.*
